# Administrative and Claims Data Help Predict Patient Mortality in Intensive Care Units by Logistic Regression: A Nationwide Database Study

**DOI:** 10.1155/2020/9076739

**Published:** 2020-02-25

**Authors:** Yu-Ting Hsu, Yi-Ting He, Chien-Kun Ting, Mei-Yung Tsou, Gau-Jun Tang, Christy Pu

**Affiliations:** ^1^Department of Anesthesiology, Taipei Veterans General Hospital, and School of Medicine, National Yang Ming University, No. 201, Section 2, Shih Pai Road, Taipei 112, Taiwan; ^2^Institute of Hospital and Health Care Administration, School of Medicine, National Yang-Ming University, No. 155, Section 2, Linong Street, Taipei 112, Taiwan; ^3^Surgical Intensive Care Unit, National Yang-Ming University Hospital, No. 169, Xiaoshe Road, Yilan City, Yilan County 260, Taiwan

## Abstract

**Background:**

Increasing attention has been paid to the predictive power of different prognostic scoring systems for decades. In this study, we compared the abilities of three commonly used scoring systems to predict short-term and long-term mortalities, with the intention of building a better prediction model for critically ill patients. We used the data from the National Health Insurance Research Database (NHIRD) in Taiwan, which included information on patient age, comorbidities, and presence of organ failure to build a new prediction model for short-term and long-term mortalities.

**Methods:**

We retrospectively collected the medical records of patients in the intensive care unit of a regional hospital in 2012 and linked them to the claims data from the NHIRD. The Acute Physiology and Chronic Health Evaluation II (APACHE II) score, Elixhauser Comorbidity Index (ECI), and Charlson Comorbidity Index (CCI) were compared for their predictive abilities. Multiple logistic regression tests were performed, and the results were presented as receiver operating characteristic curves and C-statistic.

**Results:**

The APACHE II score has the best predictive power for inhospital mortality (0.79; C − statistic = 0.77 − 0.83) and 1-year mortality (0.77; C − statistic = 0.74 − 0.79). The ECI and CCI alone have poorer predictive power and need to be combined with other variables to be comparable to the APACHE II score, as predictive tools. Using CCI together with age, sex, and whether or not the patient required mechanical ventilation is estimated to have a C-statistic of 0.773 (95% CI 0.744-0.803) for inhospital mortality, 0.782 (95% CI 0.76-0.81) for 30-day mortality, and 0.78 (95% CI 0.75-0.80) for 1-year mortality.

**Conclusions:**

We present a new prognostic model that combines CCI with age, sex, and mechanical ventilation status and can predict mortality, comparable to the APACHE II score.

## 1. Introduction

Intensive care units (ICUs) provide crucial medical care to critically ill patients. Owing to the advances in diagnostics and therapeutics, human life expectancy has been extending, leading to increasing demand for ICU care [1]. The increase in ICU care is accompanied by a growing use of risk assessment tools, aimed at evaluating treatment [2], triage patients [3], and achieve better resource allocation [4].

In the past decades, several risk scoring systems have been introduced [5]. These include the Acute Physiology and Chronic Health Evaluation (APACHE) II score [6], Charlson Comorbidity Index (CCI) [7–9], and Elixhauser Comorbidity Index (ECI) [10, 11]. However, the predictive power of these scoring systems varies according to previous surveys, and how to choose the best system is still not clear.

This study compares the abilities of these three scoring systems to predict short-term and long-term mortalities by combining data from ICU medical records with claims data from Taiwan's National Health Insurance Research Database (NHIRD) [12]. In this study, we have examined and evaluated the different variables using multiple logistic regression tests to identify and compare the strongest predictors. We aim to improve the predictive power of the current scoring systems and to build a new prediction model for ICU mortality.

## 2. Methods

### 2.1. Data Acquisition and Extraction

All ICU admissions (*n* = 2201) in 2012 at the National Yang-Ming University Hospital, a regional hospital in Eastern Taiwan, were identified. The study was approved by the Institutional Review Board of the National Yang-Ming University Hospital (IRB number: 2014A021). Informed consent was obtained from the patients or their guardians for all data used in this study.

The enrolled patients were admitted to our ICU between January 1 and December 31, 2012. If a patient was admitted more than once during the study period, only the first admission was included to avoid a small group of patients dominating the characteristics of the study population. As a result, 591 repeat admissions were excluded. Whether some of the remaining patients had been admitted to an ICU other than ours could not be determined because we lacked access to the medical records of other hospitals. We also excluded the following patients: (a) those who did not have a Taiwanese citizenship, as noncitizens did not have the national identification number required to link the ICU medical records to the claims and mortality data of NHIRD; (b) those who were under 20 years of age; and (c) those whose data could not be linked to NHIRD because of administrative errors (*n* = 1). Finally, we enrolled 1,608 patients in our study.

The ICU medical records were linked to the claims data of NHIRD from 2010 to 2013. The Taiwan NHI program is a public insurance system in which the enrollment is compulsory for Taiwanese citizens [12]. We used the patients' national identification numbers for linkage to the NHIRD in our study. Doing so reduced the likelihood of duplication and mismatching. The medical claims for all inpatient and outpatient medical services in Taiwan are the same. The medical claim and diagnosis codes in the NHI system are used nationwide and have been validated by the NHI Administration of Taiwan [13].

### 2.2. Study Variables

#### 2.2.1. Acute Physiology and Chronic Health Evaluation (APACHE) II Score

Each patient admitted to the ICU was evaluated using an APACHE II score form, which included 12 items (Table 1). These items assessed the acute physiological state, age, and chronic health conditions of the patient [6].

#### 2.2.2. Charlson Comorbidity Index (CCI)

The CCI was calculated based on 17 disease categories [7] (Table 1). The look-back period for comorbidities was 1 year before ICU admission. A 1-year look-back period is thought to improve the ability of a model to predict posthospitalization mortality according to previous studies [14, 15]. A patient was considered to have a comorbid condition in a certain year if there were at least two claim records with an ICD-9 code for that condition during that year [15, 16]. A higher CCI score indicates a higher number of comorbidities. See [Supplementary-material supplementary-material-1] for the ICD-9-CM codes for the different comorbidities [11].

#### 2.2.3. Elixhauser Comorbidity Index (ECI)

The ECI was based on 30 comorbidities [10] (Table 1). [Supplementary-material supplementary-material-1] lists the ICD-9-CM codes for these conditions [11].

#### 2.2.4. Other Variables

The other variables evaluated in the study were age, sex, hemodialysis, surgery, number of outpatient and emergency department visits in the previous year, number of inpatient admissions in the previous year, admissions department, and use of a ventilator.

### 2.3. Outcomes

The primary outcome measured in this study was inhospital mortality, which was defined as death during the hospital stay; the censoring point was discharge from the hospital. The secondary outcomes were all-cause 30-day mortality (defined as death within 30 days after hospital discharge) and overall 1-year mortality (defined as death within 1 year after hospital discharge).

### 2.4. Statistical Analysis

Data were expressed as absolute numbers and percentages. The categorical variables of surviving and deceased subjects were compared using the chi-square test. To evaluate the risk of mortality, an odds ratio with a 95% confidence interval (CI) was determined for each variable via linear regression analysis. We developed regression models to identify the strongest predictors of mortality, which were then entered into multiple logistic regression models to evaluate the overall model performance and to predict the risk of mortality. We computed the areas under receiver operating characteristic (ROC) curves (AUROCs) [17, 18] as a measure of the ability of a model to predict mortality over different risk categories. The AUROC is often referred to as the concordance index number (C-statistic) and ranges from 0.5 (no discrimination) to 1.0 (perfect discrimination), with values above 0.7, 0.8, and 0.9 considered reasonable, strong, and exceptional, respectively [18].

The discrimination performance of the CCI and ECI (with/without additional variables) was compared to that of the APACHE II score, which was used as the reference model. Differences in the AUROC between the fitted models were analyzed. Statistical analysis was performed using the SPSS statistical software (SPSS Inc., Chicago, IL, USA).

## 3. Results

The patient (*n* = 1608) characteristics stratified by inhospital mortality have been summarized in Table 2. The inhospital mortality rate was 16.11%. The inhospital mortality and no inhospital mortality groups differed significantly in terms of age, department, use and duration of the use of a ventilator, number of inpatient admissions, and CCI, ECI, and APACHE II scores.

Tables 3 and 4 show the results of univariate analyses (odds ratios, 95% confidence intervals (CIs), and *p* values) assessing the association between mortality and the CCI and ECI, respectively, alone and in combination with other variables.

The predictive abilities of the CCI and ECI, along with other variables, as represented by the C-statistic are shown in Tables 5 and 6, respectively. For inhospital mortality, the predictive power of the APACHE II score was 0.79 (95% CI: 0.77–0.82), whereas that of the CCI alone was 0.61 (95% CI: 0.57–0.64). The C-statistic for inhospital mortality based on CCI increased to 0.77 (95% CI: 0.74–0.80) when age, sex, and mechanical ventilation were added (CCI: MODEL 2) and further increased to 0.79 (95% CI: 0.76–0.82) when all variables were included (CCI: MODEL 1). The APACHE II score had a higher predictive power for 30-day mortality (C-statistic: 0.80, 95% CI: 0.78–0.82) than did the CCI alone (C-statistic: 0.62, 95% CI: 0.59–0.66).

The ECI as the sole independent variable had a lower predictive power for inhospital, 30-day, and 1-year mortality than that of the APACHE II score and CCI. It had a C-statistic of 0.55 (95% CI: 0.52–0.59) for inhospital mortality, which increased slightly when age, sex, and mechanical ventilation were added to the model (ECI: MODEL 2). It was highest (0.78, 95% CI: 0.75–0.80) when all variables were included (ECI: MODEL 1). The ECI alone had a predictive power of 0.57 (95% CI: 0.54–0.61) for 30-day mortality and 0.62 (95% CI: 0.59–0.65) for 1-year mortality.

**Figure 1:**
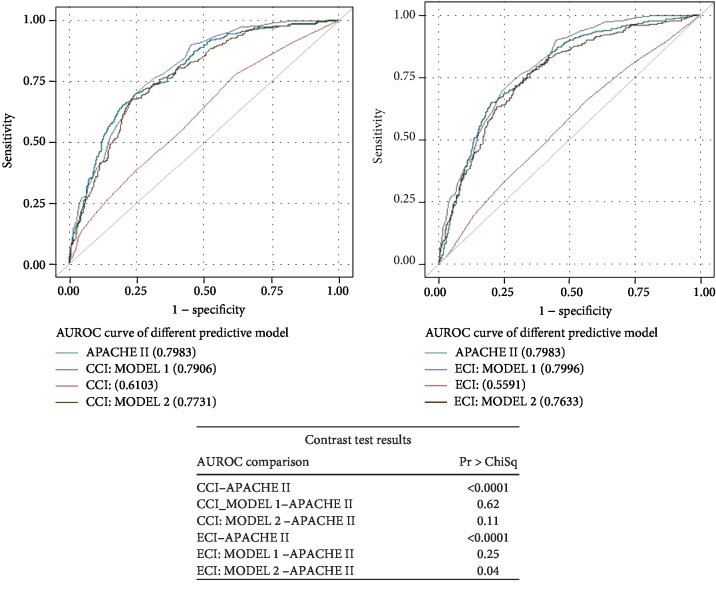
ROC curve for predicting inhospital mortality and the contrast test results. Shown are the ROC curves for (a) APACHE II score, CCI, and CCI: MODELS 1 and 2 and (b) APACHE II score, ECI, and ECI: MODELS 1 and 2.

**Figure 2:**
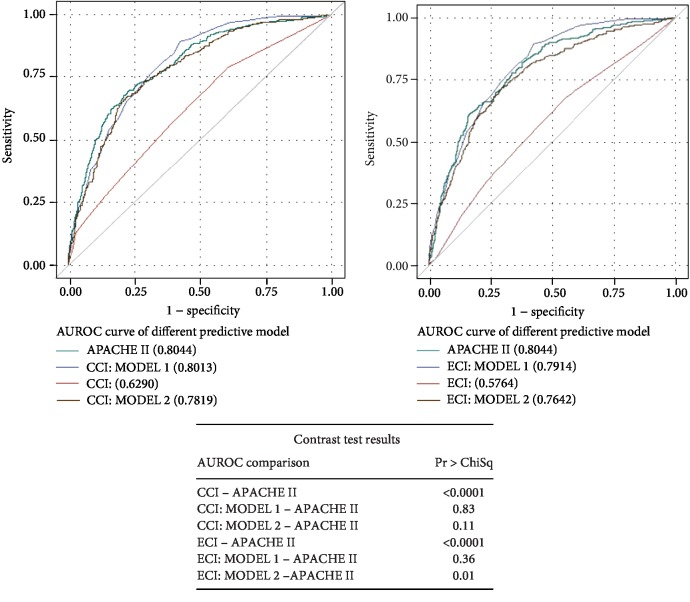
ROC curve for predicting 30-day mortality and the contrast test results. Shown are the ROC curves for (a) APACHE II score, CCI, and CCI: MODELS 1 and 2 and (b) APACHE II score, ECI, and ECI: MODELS 1 and 2.

**Figure 3:**
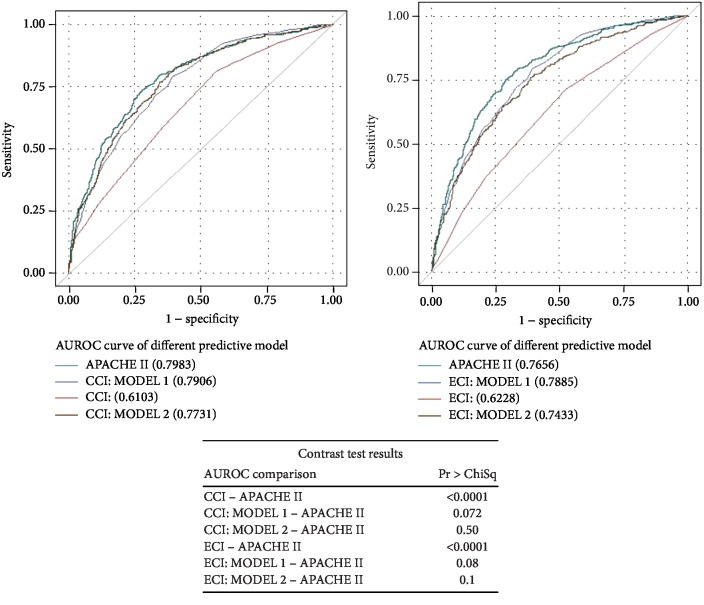
ROC curve for predicting 1-year mortality and the contrast test results. Shown are the ROC curves for (a) APACHE II score, CCI, and CCI: MODELS 1 and 2 and (b) APACHE II score, ECI, and ECI: MODELS 1 and 2.

Figures 1–3 present the analysis of the AUROC for the prediction of inhospital, 30-day, and 1-year mortality. Contrast tests revealed that the APACHE II score and CCI (CCI MODEL 1) had comparably predicted the three mortality outcomes.

## 4. Discussion

This study investigated and compared the predictive power of the APACHE II score, CCI, and ECI for short-term and long-term mortalities in ICU patients. The impact of comorbidities on predictive ability was analyzed using administrative data, which are more accurate than are ICU medical records. Our results show that the CCI and ECI have less predictive power than does the APACHE II score. However, when the CCI was combined with age, sex, and ventilator use, its ability to predict mortality increased and was comparable to that of the APACHE II score. The ECI had a poorer predictive power than that of the CCI, even when combined with age, sex, and mechanical ventilation. However, further adding the number of outpatient visits, emergency department visits, and inpatient admissions during the past year improved its predictive power slightly.

The APACHE II score has been used worldwide for measuring ICU performance. The scoring system was validated and outlined in 1985 by Knaus et al. [6] and is still a popular prognosis evaluation tool in ICU settings [19]. The APACHE II score takes into account various parameters, including acute physiological variables and chronic health conditions, all of which have significant effects on the outcome prediction for ICU patients. The CCI was introduced in 1987 to predict 1-year mortality using comorbidity data from medical charts [7]. The ECI was developed using patient data from 438 acute care hospitals in California in 1992 [10], and its outcome measures were selected from those commonly available in administrative databases.

Previous studies have investigated the predictive power of the various scoring systems and their ability to determine mortality rates using administrative data. Quach et al. [20] compared the discriminative ability of the CCI and APACHE II score in predicting hospital mortality in adult multisystem ICU patients and found the former to be less effective. In their study, the CCI did not provide significantly better results even when adjusted for age, sex, and acute physiology score. However, its predictive power slightly improved when it was added to the full APACHE II model. Despite its underperformance, the CCI can be considered an alternative method of risk assessment when data for the variables included in the APACHE II score are unavailable or not recorded in a standard manner. Christensen et al. [21] studied 469 adult patients admitted to a tertiary university-affiliated ICU and found that there were no major differences in predictive power for mortality between physiology-based systems and the CCI combined with other administrative data. Fortin et al. [22] studied the predictive performance of the ECI for inhospital mortality in adult patients at a health center and found that it demonstrated excellent discrimination for all-cause inhospital mortality. Comorbidity indices and APACHE II scores have also been used to study the severity of and mortality risk adjustments for specific health conditions such as ischemic stroke [23], acute intracerebral hemorrhage [24], trauma [25], and cancer [26].

Previous studies that enrolled different types of patients have shown that the APACHE II score has the highest predictive power for mortality compared with other comorbidity indices [20, 27, 28]. These studies usually focused on short-term mortality, and studies on longer-term mortality are limited [29].

One possible reason for the significantly higher predictive power of the APACHE II score for mortality in ICU patients when compared to that of the CCI and ECI is that the acute physiology status is usually more critical in ICU patients than in other patients and varies significantly among patients. Our study results are similar to those of Ho et al. [27], who showed that replacing the chronic condition measures of the APACHE II score with those of the CCI or ECI did not significantly improve the mortality-predicting power of the APACHE II score. On the other hand, Quach et al. [20] found that if the CCI was combined with the APACHE II score, its predictive power increased from 0.626 to 0.74. Our study shows that acute physiology variables should not be replaced by other comorbidity measures when predicting mortality in ICU patients.

When comparing short-term and long-term mortalities, we found that the APACHE II score had a slightly better predictive power for short-term mortality, whereas the CCI had a higher predictive power for long-term mortality. However, the predictive power of the CCI for long-term mortality was still lower than that of the APACHE II score. The poorer performance of the CCI may be because of its coarse weights. If we put the comorbidity variables of the CCI in a regression model as a 0–1 binary indicator, the predictive ability of the CCI may strengthen. Like the CCI, the ECI also predicted long-term mortality better than short-term mortality. This supports our conclusion that acute physiology status is more important for short-term versus long-term mortality. While the APACHE II score was originally designed to measure the severity of the conditions in critical care patients, the CCI and ECI were not. This probably explains the higher predictive power of the APACHE II score in our study [21, 28]. However, considering the time and cost involved in data collection, comorbidity measures derived from administrative data (such as those used in the CCI) may still have substantial advantages in terms of data accessibility [30].

## 5. Limitations

This study has some limitations. First, this study retrospectively collected patient information from a single regional hospital in Eastern Taiwan. Considering the limited study period and single geographic location, our findings cannot be extrapolated to other ICUs in Taiwan. A study design including different hospitals would, therefore, produce more reliable data. Second, our findings need to be validated by prospective analysis of subsequent ICU admissions. Finally, although the use of the NHIRD has advantages (e.g., large sample sizes, long observation periods, updated information, and easy access to different information sources), it also has some disadvantages, such as possible misclassification of diseases and difficulty in controlling confounding factors [31].

## 6. Conclusions

For ICU patients, the APACHE II score has the strongest predictive power for short-term mortality, followed in turn by the CCI and ECI. The ability of our new model, which combines the CCI with age, sex, and use of mechanical ventilation, to predict short-term and long-term mortality in ICU patients is comparable to that of the APACHE II score.

## Figures and Tables

**Table 1:** Variables included in the scoring systems.

APACHE	CCI	ECI
Physiologic variables Body temperature Mean arterial pressure Heart rate Respiratory rate Oxygenation Arterial pH Serum sodium Serum potassium Serum creatinine Hematocrit White blood cell count Glasgow Coma Scale Chronic health condition History of severe organ system insufficiency or immunocompromise: (a) Nonoperative or emergent postoperative patients (b) Elective postoperative patients Age	Score = 1 Myocardial infarction (history of, not just ECG changes) Congestive heart failure Peripheral disease (includes aortic aneurysms ≥ 6 cm) Cerebrovascular disease: TIA or mild or residual CVA Dementia Chronic pulmonary disease Connective tissue disease Peptic ulcer disease Mild liver disease (without portal hypertension, includes chronic hepatitis) Diabetes without end-organ damage (excludes diabetes controlled by diet alone) Score = 2 Hemiplegia Moderate or severe renal disease Diabetes with end-organ damage (retinopathy, neuropathy, nephropathy, or brittle diabetes) Nonmetastatic tumor without metastasis (exclude if >5 y from diagnosis) Leukemia (acute or chronic) Lymphoma Score = 3 Moderate or severe liver disease Score = 6 Metastatic solid tumor AIDS (not just HIV-positive)	Congestive heart failure Cardiac arrhythmias Valvular heart disease Pulmonary circulation disorders Peripheral vascular disorders Hypertension Paralysis Other neurological disorders Chronic pulmonary disease Diabetes (uncomplicated) Diabetes (complicated) Hypothyroidism Renal failure Liver disease Peptic ulcer disease excluding bleeding AIDS Lymphoma Metastatic cancer Solid tumor without metastasis Rheumatoid arthritis/collagen coagulopathy Obesity Weight loss Fluid and electrolyte disorders Blood loss anemia Deficiency anemia Alcohol abuse Drug abuse Psychoses Depression

Abbreviations: APACHE: Acute Physiology and Chronic Health Evaluation; CCI: Charlson Comorbidity Index; ECI: Elixhauser Comorbidity Index; ECG: electrocardiogram; CVA: cerebrovascular accident; TIA: transient ischemic attack; AIDS: acquired immunodeficiency syndrome; HIV: human immunodeficiency virus.

**Table 2:** Characteristics of 1608 adult ICU patients stratified by inhospital mortality.

Characteristic	Inhospital mortality		*p* value
	No	Yes	
Sex			0.2993
Female	534 (39.58)	112 (43.24)	
Male	815 (60.42)	147 (56.76)	
Age (years)	65.23 ± 18.05	74.02 ± 4.75	<0.0001^∗∗∗^
≤44	198 (14.68)	16 (6.18)	
45–64	359 (26.61)	34 (13.13)	
65–84	639 (47.37)	138 (53.28)	
≥85	153 (11.34)	71 (27.41)	
Department			0.0015^∗∗^
Internal medicine	839 (62.19)	188 (72.59)	
Surgical	510 (37.81)	71 (27.41)	
Operation			0.0633
No	987 (73.17)	204 (78.76)	
Yes	362 (26.83)	55 (21.24)	
Hemodialysis			0.5204
No	1250 (92.66)	237 (91.51)	
Yes	99 (7.34)	22 (8.49)	
Mechanical ventilation			<0.0001^∗∗∗^
No	872 (64.64)	63 (24.32)	
Yes	477 (35.36)	196 (75.68)	
ICU length of stay (days)	3.99 ± 4.44	4.25 ± 4.75	0.0716
1–7	1179 (87.40)	215 (83.01)	
>7	170 (12.60)	44 (16.99)	
Length of mechanical ventilation (days)			<0.0001^∗∗∗^
0	872 (64.64)	63 (24.32)	
1–7	319 (23.65)	152 (58.69)	
>7	158 (11.71)	44 (16.99)	
Number of outpatient visits	34.74 ± 28.24	36.78 ± 24.27	0.0892
0–15	385 (28.54)	57 (22.01)	
15–30	320 (23.72)	64 (24.71)	
>31	644 (47.74)	138 (53.28)	
Number of inpatient visits	2.12 ± 2.00	(2.61 ± 2.33)	0.0002^∗∗^
1	737 (54.63)	108 (41.70)	
2	289 (21.42)	61 (23.55)	
>3	323 (23.94)	90 (34.75)	
CCI	2.49 ± 2.11	3.52 ± 2.83	<0.0001^∗∗∗^
0	233 (17.27)	24 (9.27)	
1	282 (20.90)	33 (12.74)	
2	278 (20.61)	61 (23.55)	
≥3	556 (41.22)	141 (54.44)	
ECI	3.10 ± 1.32	3.46 ± 2.15	0.048^∗^
0	178 (13.19)	28 (10.81)	
1	182 (13.49)	24 (9.27)	
2	221 (16.38)	36 (13.90)	
3	768 (56.93)	171 (66.02)	
APACHE II score	13.80 ± 7.97	23.27 ± 8.20	<0.0001^∗∗∗^
0–14	773 (57.30)	40 (15.44)	
15–25	460 (34.10)	128 (49.42)	
≥26	116 (8.60)	91 (35.14)	

Data are expressed as *n* (%) or mean ± standard deviation. ^∗^<0.05; ^∗∗^<0.01; ^∗∗∗^<0.001. Abbreviations: ICU: intensive care unit; APACHE II: Acute Physiology and Chronic Health Evaluation II; CCI: Charlson Comorbidity Index.

**Table 3:** Univariate analysis assessing the association between the CCI with/without additional variables and mortality.

Categorical variable	OR	95% CI	*p* value
CCI	1.18	(1.10–1.26)	<0.0001^∗∗∗^
Sex	0.95	(0.71–1.28)	0.736
Age	1.03	(1.02–1.04)	<0.0001^∗∗∗^
Division	1.09	(0.74–1.60)	0.658
Operation	0.48	(0.31–0.73)	0.001^∗∗^
Hemodialysis	1.36	(0.77–2.39)	0.291
Mechanical ventilation	6.53	(4.72–9.03)	<0.0001^∗∗∗^
Number of outpatient visits	0.99	(0.99–0.99)	0.008^∗∗^
Number of inpatient visits	1.01	(0.94-1.08)	0.863

^∗^<0.05; ^∗∗^<0.01; ^∗∗∗^<0.001. Abbreviations: CCI: Charlson Comorbidity Index; OR: odds ratio; CI: confidence interval.

**Table 4:** Univariate analysis assessing the association of the ECI with/without additional variables and mortality.

Categorical variable	OR	95% CI	*p* value
ECI	0.98	(0.90–1.07)	0.708
Sex	0.90	(0.67–1.22)	0.498
Age	1.03	(1.02–1.04)	<0.0001^∗∗∗^
Division	1.20	(0.82–1.76)	0.356
Operation	0.52	(0.34–0.79)	0.002^∗∗^
Hemodialysis	1.52	(0.87–2.66)	0.146
Mechanical ventilation	6.33	(4.59–8.72)	<0.0001^∗∗∗^
Number of outpatient visits	1.00	(0.99–1.00)	0.169
Number of inpatient visits	1.08	(1.01–1.16)	0.024^∗^

^∗^<0.05; ^∗∗^<0.01; ^∗∗∗^<0.001; Abbreviations: ECI: Elixhauser Comorbidity Index; OR: odds ratio; CI: confidence interval.

**Table 5:** Predictive ability of the CCI combined with selected variables according to the C-statistic.

Score/measure	Inhospital mortality	30-day mortality	1-year mortality
Mortality (number, %)	(259, 16.11%)	(337, 20.96%)	(518, 32.21%)
APACHE II (C-statistic, 95% CI)	0.79 (0.77–0.82)	0.80 (0.78–0.82)	0.76 (0.74–0.79)
CCI (C-statistic, 95% CI)			
Alone	0.61 (0.57–0.64)	0.62 (0.59–0.66)	0.67 (0.64–0.70)
+Age, sex	0.67 (0.64–0.71)	0.69 (0.66–0.72)	0.73 (0.71–0.76)
+Age, sex, MV (CCI: MODEL 2)	0.77 (0.74–0.80)	0.78 (0.75–0.80)	0.77 (0.75–0.79)
+Age, sex, MV, OP	0.78 (0.75–0.81)	0.79 (0.77–0.82)	0.78 (0.76–0.81)
+Age, sex, MV, OP, outvisits	0.79 (0.76–0.82)	0.79 (0.77–0.82)	0.78 (0.76–0.81)
+Age, sex, MV, OP, outvisits, dept	0.79 (0.76–0.81)	0.80 (0.77–0.82)	0.78 (0.76–0.81)
+Age, sex, MV, OP, outvisits, dept, H/D	0.79 (0.76–0.81)	0.80 (0.77–0.82)	0.78 (0.76–0.81)
+ age, sex, MV, OP, out-visits, dept, H/D, invisits (CC: MODEL 2)	0.79 (0.76–0.82)	0.80 (0.77–0.82)	0.80 (0.77–0.82)

Abbreviations: APACHE II: Acute Physiology and Chronic Health Evaluation II; CCI: Charlson Comorbidity Index; MV: mechanical ventilation; CI: confidence interval; OP: operation; outvisits: number of outpatient visits; dept: department; H/D: hemodialysis; invisits: number of inpatient visits. The CCI combined with age, sex, and MV was named as CCI: MODEL 2. The CCI combined with age, sex, MV, OP, outvisits, dept, H/D, and invisits was named as CCI: MODEL 1.

**Table 6:** The predictive ability of the ECI combined with selected variables according to the C-statistic.

Score/measure	Inhospital mortality	30-day mortality	1-year mortality
Mortality (number, %)	(259, 16.11%)	(337, 20.96%)	(518, 32.21%)
APACHE II (C-statistic, 95% CI)	0.79 (0.77–0.82)	0.80 (0.78–0.82)	0.76 (0.74–0.79)
ECI			
Alone	0.55 (0.52–0.59)	0.57 (0.54–0.61)	0.62 (0.59–0.65)
+Age, sex	0.65 (0.62–0.69)	0.66 (0.63–0.70)	0.70 (0.67–0.72)
+Age, sex, MV (ECI: MODEL 2)	0.76 (0.73–0.79)	0.76 (0.73–0.79)	0.74 (0.71–0.76)
+Age, sex, MV, OP	0.77 (0.74–0.80)	0.77 (0.75–0.80)	0.75 (0.73–0.78)
+Age, sex, MV, OP, outvisits	0.77 (0.74–0.80)	0.78 (0.76–0.81)	0.78 (0.76–0.80)
+Age, sex, MV, OP, outvisits, dept	0.77 (0.74–0.80)	0.78 (0.76–0.81)	0.78 (0.76–0.81)
+Age, sex, MV, OP, outvisits, dept, H/D	0.77 (0.74–0.80)	0.78 (0.76–0.81)	0.78 (0.76–0.81)
+Age, sex, MV, OP, outvisits, dept, H/D, invisits (ECI: MODEL 1)	0.78 (0.75–0.80)	0.79 (0.76–0.81)	0.78 (0.76–0.81)

Abbreviations: APACHE II: Acute Physiology and Chronic Health Evaluation II; ECI: Elixhauser Comorbidity Index; MV: mechanical ventilation; OP: operation; outvisits: number of outpatient visits; dept: department; H/D: hemodialysis; invisits: number of inpatient visit. The ECI combined with age, sex, and MV was named as ECI: MODEL 2. The ECI combined with age, sex, MV, OP, outvisits, dept, H/D, and invisits was named as ECI: MODEL 1.

## Data Availability

The data sets used and analyzed in this study are available from the corresponding author on reasonable request.

## Abbreviations

APACHE: Acute Physiology and Chronic Health Evaluation
AUROC: Area under the receiver operating characteristic curve
CCI: Charlson Comorbidity Index
CI: Confidence interval
ECI: Elixhauser Comorbidity Index
ICU: Intensive care unit
NHI: National Health Insurance
NHIRD: National Health Insurance Research Database
OR: Odds ratio
ROC: Receiver operating characteristic.

## Supplementary Materials

Supplementary Materials ICD-9 codes for different comorbidities evaluated in the study.

## References

[1] Gooch R. A., Kahn J. M. (2014). ICU bed supply, utilization, and health care spending: an example of demand elasticity. *JAMA*.

[2] Mery E., Kahn J. M. (2013). Does space make waste? The influence of ICU bed capacity on admission decisions. *Critical Care*.

[3] Stelfox H. T., Hemmelgarn B. R., Bagshaw S. M. (2012). Intensive care unit bed availability and outcomes for hospitalized patients with sudden clinical deterioration. *Archives of Internal Medicine*.

[4] Chen L. M., Render M., Sales A., Kennedy E. H., Wiitala W., Hofer T. P. (2012). Intensive care unit admitting patterns in the veterans affairs health care system. *Archives of Internal Medicine*.

[5] Sharabiani M. T., Aylin P., Bottle A. (2012). Systematic review of comorbidity indices for administrative data. *Medical Care*.

[6] Knaus W. A., Draper E. A., Wagner D. P., Zimmerman J. E. (1985). APACHE II: a severity of disease classification system. *Critical Care Medicine*.

[7] Charlson M. E., Pompei P., Ales K. L., MacKenzie C. (1987). A new method of classifying prognostic comorbidity in longitudinal studies: development and validation. *Journal of Chronic Diseases*.

[8] Poses R. M., McClish D., Smith W. R., Bekes C., Scott W. E. (1996). Prediction of survival of critically ill patients by admission comorbidity. *Journal of Clinical Epidemiology*.

[9] Needham D. M., Scales D. C., Laupacis A., Pronovost P. J. (2005). A systematic review of the Charlson comorbidity index using Canadian administrative databases: a perspective on risk adjustment in critical care research. *Journal of Critical Care*.

[10] Elixhauser A., Steiner C., Harris D. R., Coffey R. M. (1998). Comorbidity measures for use with administrative data. *Medical Care*.

[11] Quan H., Sundararajan V., Halfon P. (2005). Coding algorithms for defining comorbidities in ICD-9-CM and ICD-10 administrative data. *Medical Care*.

[12] Cheng T. M. (2003). Taiwan's new national health insurance program: genesis and experience so far. *Health Affairs*.

[13] Cheng C. L., Kao Y. H., Lin S. J., Lee C. H., Lai M. L. (2011). Validation of the National Health Insurance Research Database with ischemic stroke cases in Taiwan. *Pharmacoepidemiology and Drug Safety*.

[14] Preen D. B., Holman C. D., Spilsbury K., Semmens J. B., Brameld K. J. (2006). Length of comorbidity lookback period affected regression model performance of administrative health data. *Journal of Clinical Epidemiology*.

[15] Deyo R. A., Cherkin D. C., Ciol M. A. (1992). Adapting a clinical comorbidity index for use with ICD-9-CM administrative databases. *Journal of Clinical Epidemiology*.

[16] Norena M., Wong H., Thompson W. D., Keenan S. P., Dodek P. M. (2006). Adjustment of intensive care unit outcomes for severity of illness and comorbidity scores. *Journal of Critical Care*.

[17] Pencina M. J., D'Agostino R. B., Vasan R. S. (2010). Statistical methods for assessment of added usefulness of new biomarkers. *Clinical Chemistry and Laboratory Medicine*.

[18] Hanley J. A., McNeil B. J. (1983). A method of comparing the areas under receiver operating characteristic curves derived from the same cases. *Radiology*.

[19] Rué M., MD A. A., Álvarez M., Quintana S., Valero C. (2000). Performance of the Mortality Probability Models in assessing severity of illness during the first week in the intensive care unit. *Critical Care Medicine*.

[20] Quach S., Hennessy D. A., Faris P., Fong A., Quan H., Doig C. (2009). A comparison between the APACHE II and Charlson Index Score for predicting hospital mortality in critically ill patients. *BMC Health Services Research*.

[21] Christensen S., Johansen M. B., Christiansen C. F., Jensen R., Lemeshow S. (2011). Comparison of Charlson comorbidity index with SAPS and APACHE scores for prediction of mortality following intensive care. *Clinical Epidemiology*.

[22] Fortin Y., Crispo J. A., Cohen D., McNair D., Mattison D. R., Krewski D. (2017). External validation and comparison of two variants of the Elixhauser comorbidity measures for all-cause mortality. *PLoS One*.

[23] Ludwigs U., Csatlos M., Hulting J. (2000). Predicting in-hospital mortality in acute myocardial infarction: impact of thrombolytic therapy on APACHE II performance. *Scandinavian Cardiovascular Journal*.

[24] Huang Y., Chen J., Zhong S., Yuan J. (2016). Role of APACHE II scoring system in the prediction of severity and outcome of acute intracerebral hemorrhage. *The International Journal of Neuroscience*.

[25] Dossett L. A., Redhage L. A., Sawyer R. G., May A. K. (2009). Revisiting the validity of APACHE II in the trauma ICU: improved risk stratification in critically injured adults. *Injury*.

[26] Chang L., Horng C. F., Huang Y. C., Hsieh Y. Y. (2006). Prognostic accuracy of Acute Physiology and Chronic Health Evaluation II scores in critically ill cancer patients. *American Journal of Critical Care*.

[27] Ho K. M., Finn J., Knuiman M., Webb S. A. (2007). Combining multiple comorbidities with Acute Physiology Score to predict hospital mortality of critically ill patients: a linked data cohort study. *Anaesthesia*.

[28] Ho K. M., Lee K. Y., Williams T., Finn J., Knuiman M., Webb S. A. (2007). Comparison of Acute Physiology and Chronic Health Evaluation (APACHE) II score with organ failure scores to predict hospital mortality. *Anaesthesia*.

[29] Johnston J. A., Wagner D. P., Timmons S., Welsh D., Tsevat J., Render M. L. (2002). Impact of different measures of comorbid disease on predicted mortality of intensive care unit patients. *Medical Care*.

[30] Farley J. F., Harley C. R., Devine J. W. (2006). A comparison of comorbidity measurements to predict healthcare expenditures. *The American Journal of Managed Care*.

[31] Mazzali C., Duca P. (2015). Use of administrative data in healthcare research. *Internal and Emergency Medicine*.

