# Supplementary material for: Administrative and Claims Data Help Predict Patient Mortality in Intensive Care Units by Logistic Regression: A Nationwide Database Study
Source: Biomed Res Int. 2020 Feb 25;2020:9076739. doi: 10.1155/2020/9076739 (PMC7061120; doi:10.1155/2020/9076739)
Supplement: Supplementary Materials — ICD-9 codes for different comorbidities evaluated in the study. [file 9076739.f1.docx]

**Appendix 1. ICD-9-CM coding**

*Abbreviation: ICD-9, International Classification of Diseases, 9^th^ Revision*
